# Automatic Identification of Tool Wear Based on Thermography and a Convolutional Neural Network during the Turning Process

**DOI:** 10.3390/s21051917

**Published:** 2021-03-09

**Authors:** Nika Brili, Mirko Ficko, Simon Klančnik

**Affiliations:** Faculty of Mechanical Engineering, University of Maribor, Smetanova ul. 17, 2000 Maribor, Slovenia; mirko.ficko@um.si (M.F.); simon.klancnik@um.si (S.K.)

**Keywords:** tool wear, turning, infrared thermography, artificial intelligence, deep learning, Industry 4.0

## Abstract

This article presents a control system for a cutting tool condition supervision, which recognises tool wear automatically during turning. We used an infrared camera for process control, which—unlike common cameras—captures the thermographic state, in addition to the visual state of the process. Despite challenging environmental conditions (e.g., hot chips) we protected the camera and placed it right up to the cutting knife, so that machining could be observed closely. During the experiment constant cutting conditions were set for the dry machining of workpiece (low alloy carbon steel 1.7225 or 42CrMo4). To build a dataset of over 9000 images, we machined on a lathe with tool inserts of different wear levels. Using a convolutional neural network (CNN), we developed a model for tool wear and tool damage prediction. It determines the state of a cutting tool automatically (none, low, medium, high wear level), based on thermographic process data. The accuracy of classification was 99.55%, which affirms the adequacy of the proposed method. Such a system enables immediate action in the case of cutting tool wear or breakage, regardless of the operator’s knowledge and competence.

## 1. Introduction

A new technological era, called Industry 4.0, will change all manufacturing-related fields [1]. The industrial and production processes will be transformed into intelligent factory systems [2]. Production will be controlled autonomously and dynamic, with a high degree of automation [3]. Smart systems are replacing human decisions.

Due to high competition on the market and corresponding lowering of the production costs, minimal worker presence, or even unmanned machining, is becoming the key trend of the majority of manufacturing industries [4]. In manufacturing, the main source of financial and time losses arises from material waste due to machining with an excessively worn tool, and machine downtime [5].

We asked ourselves: Why are robots already replacing workers in assembly lines, at workpiece manipulation, welding, casting, etc., while turning and milling machine operators are still not (entirely) replaceable? The field of Computer Numerical Control (CNC) machine augmentation is under intensive development, which is resulting in a greater performance of the machines, capable of multiple operations on a single machine (e.g., lathes with a driven tool and an additional *y*-axis allows a milling operation), faster machining, etc. Even the latest modern machines, whose purpose is an individual or small series production, are not capable of autonomous operation without human supervision.

It is crucial for automatic, or so-called unmanned machining, to detect wear of a cutting tool edge in time to prevent negative effects on the quality of a machined surface [6,7]. Excessive cutting tool wear can also lead to serious workpiece or machine damage [8]. The wear control of a cutting tool benefits product quality enhancement, tool-related costs’ optimisation, and assists in avoiding undesired events. In small series and individual production, the machine operator is the one who determines when to change a cutting tool, based upon their experience. Bad decisions can often lead to greater costs, production downtime and scrap.

We focused on the turning process. The condition of a cutting tool affects not only workpiece properties (geometrical, surface, and structural attributes), but also the quantity of waste and frequency of production interruptions [9]. When should one change a cutting tool? As seen in Table 1, this is decided by a machine operator based on their expertise and informal knowledge (feeling, personal judgment, and experience), depending on numerous criteria [8,10].

Stochasticity of the process for a cutting tool wear determination can lead to the following scenarios:

- the cutting tool is still suitable for machining after replacement: A consequence of this is an increase of cutting tool-related costs, and time spent needlessly for tool exchange;
- the machining takes place using a worn or broken cutting tool: A consequence is a low quality of the machined surface, overheating of the workpiece and tool material, an increase of vibrations and cutting forces, which has negative effects on the machine, etc.

In practice, both scenarios occur regularly, especially due to less experienced operators, who cannot make the right decisions about a cutting tool replacement. Cutting tools’ catalogues contain cutting tool lifetime information-the time that a tool spends in contact with the workpiece. Such data would theoretically be useful if the machining process could be executed under the exact same conditions that were used during lifetime estimation. In a real environment, there are no such conditions-defects in the material, welded areas with high hardness, cutting path interruptions, uneven cooling, vibrations during machining, and other disturbances have a significant effect on the cutting tool lifetime. There is a possibility for a cutting tool to damage even at a first cut, which makes active decision-making based on constant tool supervision fundamentally better than lifetime-based decision-making.

An important influencing factor on the cutting tool wear is a tool’s thermal load. Finding correlations between machining parameters (turning, milling) and the cutting temperature is frequently an object of research [11,12,13,14,15]. There are similar conclusions:

- higher temperature causes greater tool wear, and
- cutting speed has the largest effect on the temperature.

**Figure 1:**
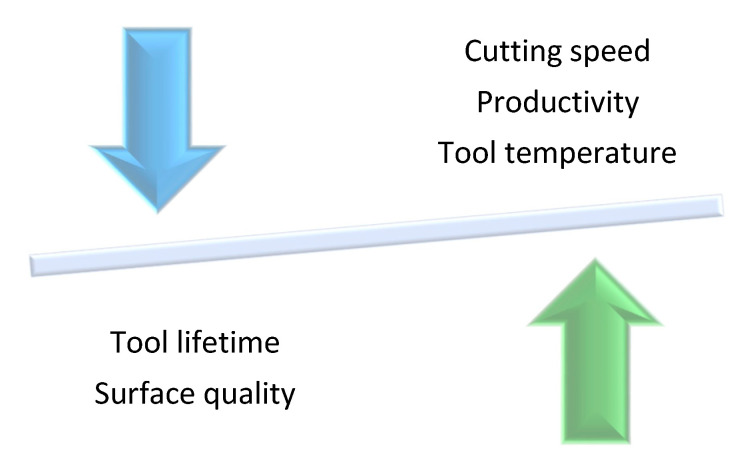
Inverse proportionality of machining parameters.

Correlations are, therefore, inversely proportional. Choosing optimal machining conditions is a significant challenge (Figure 1).

It is the interdependence of temperature and cutting tool wear that makes the method of thermography one of the possible ways for machining process control.

The optimisation of machining processes requires thorough study and comprehension of the phenomena. In 1996 an infrared (IR) camera was used to measure the temperature of chips and a cutting tool. They found that after the cutting edge is broken, the temperature of a tool rises quickly. Furthermore, there is a connection between the temperature and the wear of a tool’s flank surface [11].

The heat transfer during the turning process and lathe tool life have been researched, so that, along with the IR camera measurements, they also included thermocouple measurements, which were positioned on the tool and workpiece. A correlation has been discovered between locations on the tool with the maximum temperature and area of insert wear [12].

The IR camera measurements are ordinarily carried out laterally, i.e., perpendicular to the direction of the cutting speed, so a model was developed that calculates temperatures at the contact point between a tool and a chip. This is done based on the given process parameters (cutting force, chip thickness, tool-chip contact length) and a lateral thermal image of the tool [13,14]. It was concluded that a two-fold increase in the cutting speed (from 100 m/min to 200 m/min) causes a 20% increase in the tool temperature. Meanwhile, doubling the feed rate (from 0.1 mm/rev to 0.2 mm/rev) yields only 10% higher temperatures. All measurements were made without cooling during the machining process, due to the presence of the IR camera [13].

Another research focused on a correlation between the cutting parameters and temperature gradients on the cutting insert, where a thermal image was observed during turning (heating of insert and chips). Cutting forces were also studied, as well as chip shape and tool-chip contact length. The monitoring of the process with an IR camera was necessary to provide an adequate method for researching the mechanical and thermal aspects of cutting [15].

Many authors considered the possibilities of automatic cutting tool condition control. They found that the most suitable approach for modelling non-linear dependencies are Artificial Intelligence methods, namely artificial neural networks (ANN), fuzzy logic systems, or a hybrid of both [9,16,17,18].

Automatic prediction of the remaining life of cutting edge is possible using ANN. Accuracy of predicted flank wear is proven by conventional methods (measurements) and image recognition with the special software Neural Wear [18].

An algorithm was developed that processes the thermographic image of a tool insert: It divides it into two parts (the cutting area and surroundings), and from the cutting area discerns two temperatures that represent an input for the neural network. The temperature of the surrounding area is used to calculate the heat transfer to the rest of the tool insert. A neural network makes predictions about the temperature at the point of contact between the tool and the chip, that would otherwise be impossible to measure during turning due to physical limitations [19].

The general regression neural network (GRNN) enables a prediction of the tool’s nose wear on the cutting edge. Input data are speed, feed rate, and cutting depth. Results indicated the need for an additional three parameters (three force components) in order to get better prediction capability [20].

It was confirmed that the cutting tool flank wear affects the cutting force amplitude. Using a backpropagation neural network (BPNN), the percentage error of the predicted wear was found to be between 0.6% and 15.1%. The measured forces and parameters of turning were used as inputs for the neural network, which was comprised of 30 neurons in the hidden layer and eight neurons in the output layer (output neurons represent binary inscription of the flank wear, i.e., eight features of the wear) [21].

The insert wear can also be monitored with computer vision [22,23]. The algorithm discerns four separate wear types: wear of the flank surface, fracture, built-up edge (BUE), and chipping [23].

Another algorithm that analyses the insert, calibrates and calculates the average width and a tool wear area automatically with a 3% absolute average error [24].

Real-time tool wear and breakage detection was developed on a CNC machine. The input data were the electrical current, measured on a spindle, which was analysed with the deep learning method (a convolutional neural network with the backpropagation) [25,26].

In more recent research [27], tool wear during milling was monitored with the help of standard images and deep learning. Tools were divided into four categories of wear, each containing approximately 2000 images. Based on the database, the system learned to predict the wear with a 96.20% accuracy.

To the best of our knowledge, there are no studies that consider the control of a machining process with an IR camera and direct cutting tool condition recognition, based on a thermographic image and a prediction model. The novelty of this research is the classification model for the tool condition monitoring during machining, which achieves extremely high accuracy. The process is monitored with an IR camera which captures multiple factors simultaneously, that are shown in Table 1: Visual inspection, temperature condition, chip shape. Our research surpasses the current state-of-the-art because the proposed solution contains not only temperature measurements and assumptions based on temperature value, but a 2D-colour thermographic image, which contains substantially more features. Such an image is the bearer of a huge amount of information because each image point (i.e., pixel) is separate data, which store some absolute value. Likewise, the pixel’s location in the image is equally important, so is the arrangement of similar pixels and the differences between them, etc. Classical analytical models are not capable of decision-making based on such a large quantity of input data, therefore we developed an intelligent system, which has learned to correlate image features with the none, low, medium, and high tool wear levels. Results are highly useful for optimizing costs and processes in the manufacturing.

This paper is organised as follows: Section 2 presents the proposed method for the monitoring of the machining process and prediction model, based on the CNN; Section 3 reports the Results and Discussion; Section 4 describes the conclusions of the paper.

## 2. Materials and Methods

### 2.1. System Overview

We divided cutting tools into four categories, according to the wear level: none, low, medium, and high. The type of wear is not of interest here. Instead, we inspected the suitability of cutting tools for further turning. A similar aspect was used in the research [28] where tools were characterised into two groups: serviceable or disposable.

Each category of the cutting tool condition has been provided with more than 2000 images, which were used for compiling train and test datasets for classification model preparation.

Images were inspected and sorted into appropriate folders. The Convolutional Neural Network was trained using a training dataset, and later tested with yet-unseen test images.

The research was divided into two major sections:

- experimental part on the lathe machine (intended to build a large dataset), and
- processing of the acquired data and training/testing of the CNN model.

**Figure 2:**
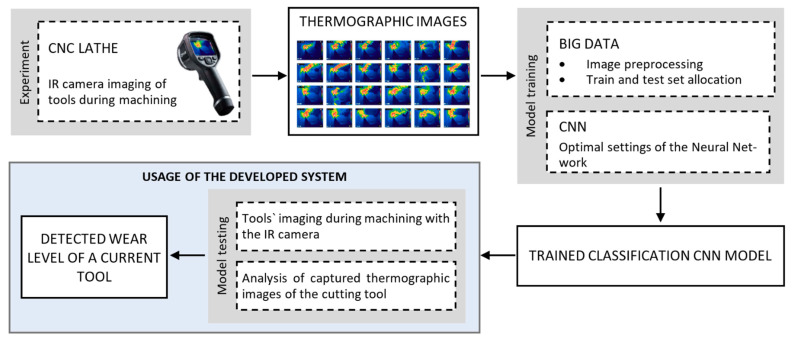
Schematic representation of the model for an intelligent control of the cutting tool wear level and breakage, using thermography.

The whole procedure of developing the tool condition monitoring model from data acquisition (thermographic images of cutting tools) up to the use of the trained model, is depicted in Figure 2.

### 2.2. Determining Cutting Tool Condition

**Figure 3:**
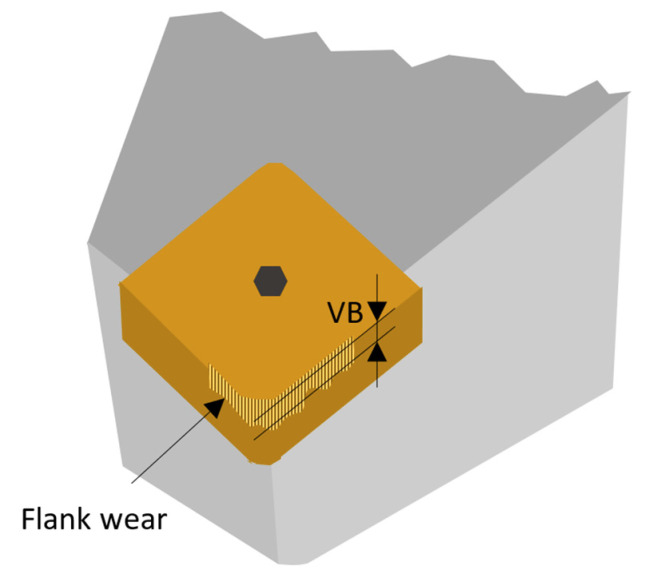
A cutting tool with the flank wear (VB).

It is proven that the flank wear grows proportionally with the machining time [29]. There are recommendations (ASTM Standard) on how to measure tool lifetime, based on the flank wear level (designation VB) or the width of the worn edge. In the case of even wear, the VB = 0.3 mm, but at uneven wear, the maximum local wear can be VB = 0.6 mm [8,30]. The meaning of VB is shown in Figure 3.

**Figure 4:**
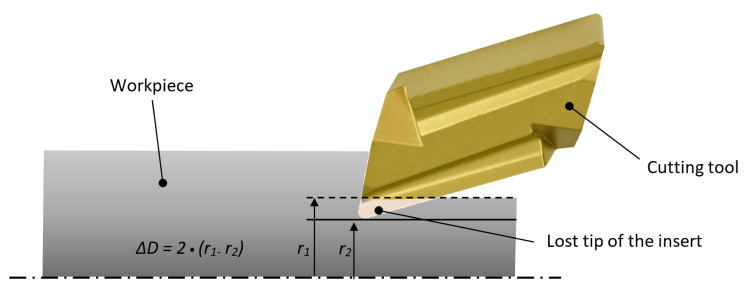
The effect of a cutting tool wear on the workpiece diameter deviation (*r_1_* is actual workpiece radius, *r_2_* is expected workpiece radius in case of no tool wear).

Some authors have pointed out the deficiency of such criteria: It does not take into account the geometry of a tool [8,31,32]. In an in-depth study [32] where the effects of the tool wear were analysed, Niaki presented an indirect method for the tool wear estimation. Total tool height changed due to wear, which leads to the dimensional deviations of the workpiece (Figure 4). Its diameter was measured after each cut at three locations. He observed how the real value deviated from the expected one (Δ*D*). The correlation between Δ*D* and flank wear VB was confirmed [32]. The method was checked and approved by comparing a predicted flank wear VB and measured deviations of the workpiece’s diameter Δ*D* at seven distinct feed speeds.

In the scope of the research we determined the cutting tool quality according to two methods:

(1) *With measurements*: Niaki’s method [32]. The method is suitable because:
  - a tool insert can stay in the fixture permanently,
  - measurements are fast, and they interrupt the machining process only momentarily (which is important for temperature monitoring, because insert cooling between individual cuts is undesirable),
  - the calibrated micrometre is the only additional equipment needed (an affordable solution),
  - the method is suitable for multiple measurements, for it is not time-consuming (a large database is required).
(2) *Experimental*: The quality of a cutting tool’s condition will be determined by an experienced expert. An algorithm will replace the expert in decision-making, therefore we want it to make decisions in the same way as the expert. They take into account all factors shown in Table 1, based on their experience.

All tool wear was concatenated into three classes, according to the intensity (low, medium and high). Altogether, this gives us 4 classification categories. Allowed deviation of the workpiece diameter due to insert wear was determined based on dimensional requirements, the diameter of the workpiece, and the tool insert type. Limit values of the wear level are written in Table 2.

### 2.3. Experiments

The experimental part of the research was designed to acquire a dataset. For this section, we set two goals we wanted to achieve:

- Acquisition of thermographic images during the machining process (turning) at maximal proximity to the cutting tool,
- Acquisition of an adequate amount of images for each distinct cutting tool’s condition.

Machining operations were executed on a CNC machine, which was adapted to suffice the aforementioned requirements. The following section presents the hardware that was used in experimentation.

#### 2.3.1. Experimental Setup

The used IR camera, a FLIR E5 (Flir Systems, Inc., Wilsonville, OR, USA), can capture nine images per second (9 Hz capture frequency), and a resolution of 120 × 90 pixels. When transferring an image to the computer, a time delay and loss occur, so the number of captured images per second was halved (4.5 image/s, or 1 image on every 0.222 s). We used the following camera settings: Rainbow scheme, without edges, emissivity: ε = 0.60. Emissivity measures how efficiently an object radiates heat. Values can be between 0 (perfect mirror that reflects all energy) and 1 (a blackbody that absorbs and radiates all energy). Value ε = 0.60 was selected according to the IR camera’s distributor recommendations based on the material of the workpiece.

**Figure 5:**
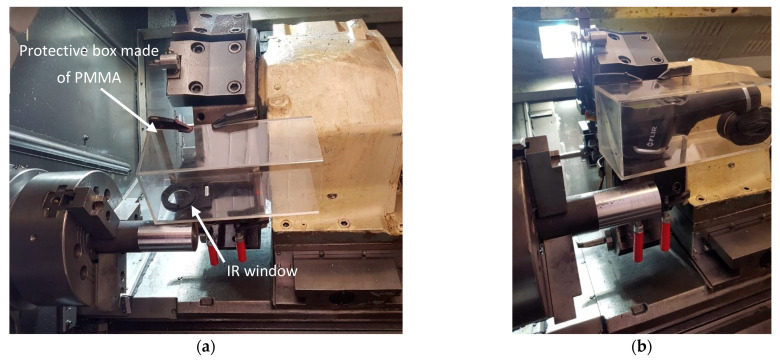
The camera position in a lathe (**a**) Protection for an infrared (IR) camera; (**b**) Mounting of the IR camera closely against the cutting knife.

The camera must be capturing images during the machining process, so it needs to be protected against hot chips. We made a polymethyl methacrylate (PMMA) box for camera protection. PMMA does not transmit IR radiation, so a hole was made in front of the lens where a custom IR window was positioned, which is both durable and IR light transmissive (Figure 5). The box with the IR camera was mounted on the revolver right up to the cutting tool-on the next tool place in the revolver. The distance between the machining location and the IR camera was less than 10 cm, so the camera moved with the cutting insert. With that, we ensured the reproducibility of image acquisition.

The experiment was executed on an Okuma LC30 CNC machine (Okuma Corporation, Aichi, Japan). The machining parameters (provided in Table 3) were selected according to the tool manufacturer’s recommendations based on the material of the workpiece, which was 1.7225 steel in the normalized state (Table 4). The initial dimension of the workpiece was Φ 60 mm, and the length of machining was 100 mm.

**Figure 6:**
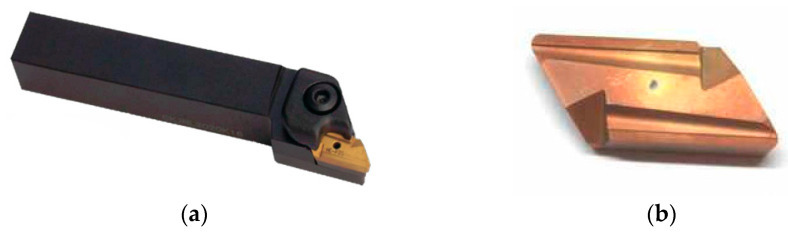
The cutting tool: (**a**) Holder of type CKJNL; (**b**) Cutting tool insert KNUX.

In the research we used a Sanstone KNUX160410L11 cutting tool (produced by Zhuzhou Yifeng Tools Co., Ltd., Hunan, China), which is the standard turning insert, made from carbide with a CVD coating (a thick, rough layer) and two cutting edges. Its main purpose is steel machining, but it also works well for machining other alloys. The cutting tool insert and holder are presented in Figure 6 and Table 5.

#### 2.3.2. Image Database

**Figure 7:**
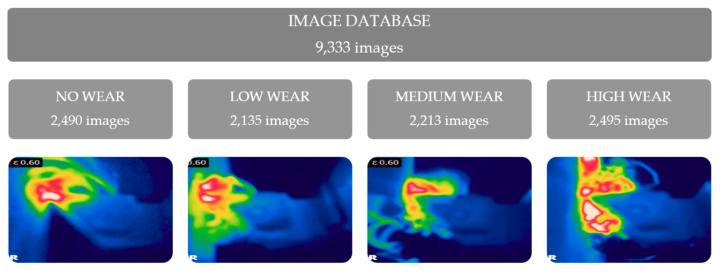
The categorisation of the acquired images into wear classes.

The acquired image database contains over 2000 images in each class of the cutting tool wear. It is portrayed in Figure 7. The detailed explanation about two methods used for determining cutting tool condition is in the Section 2.2.

As seen on the Figure 7 chips absorb the most heat generated during the cutting. At the cutting speed 100 m/min approximately 70% of the heat is absorbed by chip, 20% by tool, and 10% by workpiece [33]. On majority of the images the tool and the workpiece are not even seen due to chip locations.

The test set was created by selecting a portion of data from the training set, which the model does not have access to during training. The same number of images were selected for each class in the test set, which makes results more comparable. Nearly 10% of all images in the distinct wear class were allocated to the test set (Table 6).

### 2.4. Convolutional Neural Network

The image recognition model used in this research was Inception v3. It is Google’s pre-trained model based on a convolutional neural network, a type of deep learning neural network. Inception was originally introduced during the ImageNet Recognition Challenge. The model v3 has been shown to attain greater than 78.1% accuracy on ImageNet pictures-it was the first runner-up in the competition [34]. The model was originally trained on over a million images from 1000 classes on some very powerful machines [35].

**Figure 8:**
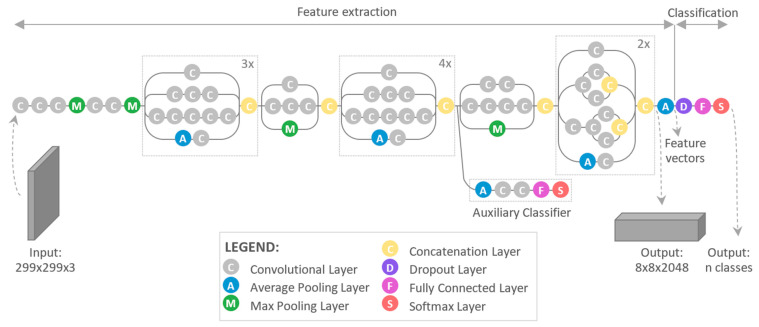
Inception V3 architecture.

Figure 8 presents the architecture of the Inception v3. The model was using loss function Softmax and was made of symmetric and asymmetric building blocks, including [36]:

- convolutions,
- average pooling,
- max pooling,
- concatenations,
- dropouts,
- fully connected layers.

**Figure 9:**
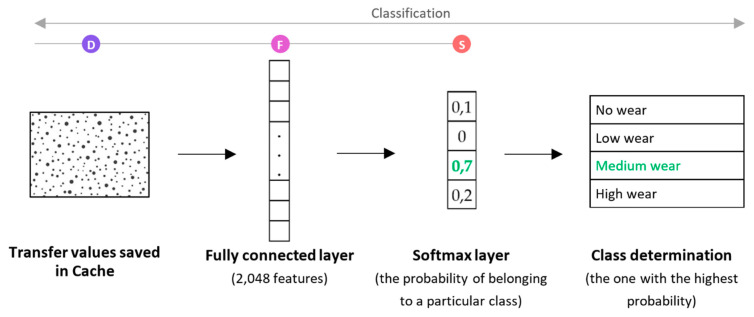
Last layers of CNN that are trained for a specific case.

We used a pre-trained Inception V3 model, which is called transfer learning - storing knowledge gained while solving one problem and applying it to a different but related problem [37]. Since the model has been pre-trained, only the last few layers which are shown on the Figure 8 must be trained on specific images (shown in Figure 9).

The Python libraries Keras and TensorFlow were used for model implementation. TensorFlow is a software framework developed by the Google Brain team. In 2019, they released version 2.0 with integrated Keras, which is a high-level API, written in Python.

### 2.5. Criteria for the Model Evaluation

Criteria for evaluating the performance of the classification were calculated using a confusion matrix, which shows the classification of images into classes according to the real situation and according to the model prediction. The matrix has the correct values written in columns and the values specified by the model in the rows (or vice versa). The correct predictions of the classifier lie on the diagonal of the matrix, and the incorrect ones outside the diagonal.

The evaluation of the results is explained in Table 7 in the case of a confusion matrix for a four-class qualification problem. The result categories are marked for Class 1, and the index j denotes the individual class. The results in the contingency matrix are marked with the following designations [32]:

- True Positive (TP). When the actual value is true, and the result of the classification model is also true;
- False Positive (FP). When the model predicted false incorrectly when the actual value was true;
- True Negative (TN). When the actual value is false and the result of the classification model is also false;
- False Negative (FN). When the classification model predicted true incorrectly when the actual value was false.

The recall [38] is calculated as the ratio between correctly classified positive cases and all true positive cases. It is calculated for each class individually. This metric indicates the correctly categorised images in each actual class of wear (the column in a confusion matrix):

(1) $$Recall=R=\frac{no.\;of\;correct\;classifications\;into\;the\;j\;class}{no.\;of\;actual\;instances\;into\;the\;j\;class}=\frac{TP}{TP+FN}$$

The precision [38] is calculated as the ratio between correctly classified positive cases and all classified positive cases. It is determined for each class separately. These are, therefore, correctly classified images in an individual classified wear class (the row in the confusion matrix):

(2) $$Precision=P=\frac{no.\;of\;correct\;classifications\;into\;the\;j\;class}{no.\;of\;all\;classifications\;into\;the\;j\;class}=\frac{TP}{TP+FP}$$

Accuracy [38] was calculated as the ratio between correctly classified cases and the number of all cases (regardless of the wear class):

(3) $$Accuracy=\frac{no.\;of\;all\;correct\;classifications}{no.\;of\;all\;classifications}=\frac{\sum_{i=1}^{j}TP_{i}}{no.\;of\;all\;classifications}$$

The evaluation indicator that can represent the performance of the model most intuitively is accuracy [39] and will be considered for the final CNN evaluation.

## 3. Results and Discussions

### 3.1. Classification of the Cutting Tool into 4 Classes

The first classification was performed using images, which are presented in Figure 7, that is for four classes of cutting tools. The results of the classification are shown in Table 8.

- Number of correct classifications 836
- Total number of images      880
- Accuracy             95.00%

The model learned to recognise various wear levels of inserts successfully, and achieved accuracy of 95%. The best recall was reached for classes “No wear” and “Low wear”; conversely, classes with the most prediction errors were “Medium wear” and “High wear”. From the point of industrial applications, it is important never to miss-categorise “High wear” to be of the class “No wear” or “Low wear”. This could lead to scrap (when using a highly worn insert for machining to the final tolerance, one would not be able to achieve the required dimensional criteria and surface roughness).

### 3.2. Determining the Optimal Number of Training Iterations

The number of training iterations is one of the most typical topological parameters of a Neural Network, that has a direct impact on the classification result quality. Generally, increasing the number of iterations causes better model accuracy, but it also increases the time needed for model training. The training time grows linearly, while the classification accuracy converges to some value. The parameter of iteration number was varied, and the CNN classification was carried out for ten distinct values of it. The number of iterations was between 10 and 10,000.

**Figure 10:**
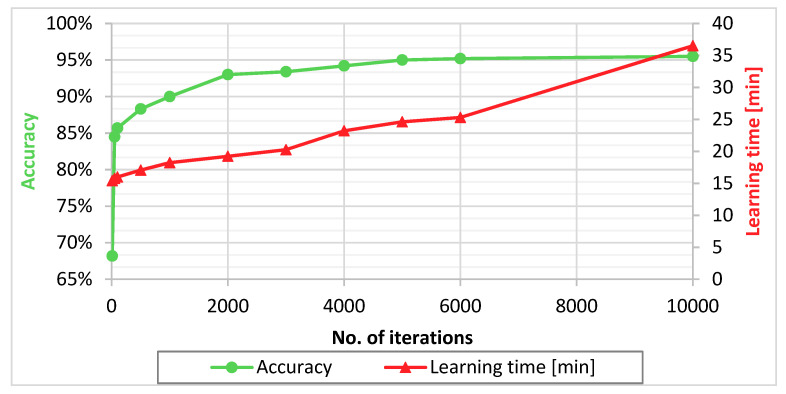
Graphical representation of the accuracy and the calculation time at different numbers of iterations.

Figure 10 shows that the accuracy of the model grows up to 5000 iterations, while the additional iterations bring a minimal, insignificant difference. 5000 iterations were made in 24.6 min and the achieved accuracy was 95.0%. At 10,000 iterations and almost 50% longer training time (36.5 min), the accuracy improved by just 0.5%. 5000 iterations have been determined as the optimal choice.

### 3.3. Classification of Cutting Tools into Three Classes

Prior results show that a CNN struggles most when classifying an insert with a medium wear level. Machining-wise and in a sense of industrial use, this category makes little sense, therefore, the decision was made to keep only three wear categories. The severity of the wear at which a tool is still usable depends on the type of workpiece’s material and its requirements (tolerance, roughness), and a general rule is written in Table 9.

The classification was repeated with 3 categories of tool wear: None, low, and high wear. The model training was performed in 5000 iterations. The results of the classification are collected in Table 10.

The classification accuracy with three classes yielded 99.55%, which is an excellent result, and confirmation of the possibility for the method to be used in a real process. Only three of 660 images were classified incorrectly. CNN made a mistake when classifying “no wear” and “low wear” tools. All images in “high wear” were predicted correctly, which is a fundamental advantage. The model could be used in industry, especially because a sudden tool breakage is the most significant factor for a smooth working process.

- Number of correct classifications 657
- Total number of images      660
- Accuracy             99.55%

### 3.4. Classifications with the Exclusion of Image Series

The nature of the experiment caused each individual image to be a part of the series (each cut of the insert represents one image series). Therefore, we made 13 different classifications, where we excluded selected image series from the training set and used it as a test set. CNN did not see any of the images from the test set during training.

**Figure 11:**
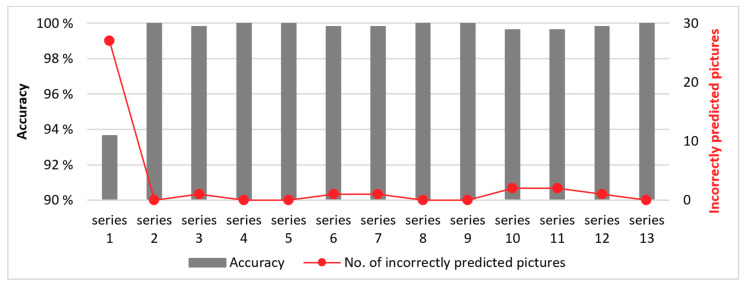
Classification results for different image test sets.

We excluded the first cut (first image series) from the training set in the first classification. The second image series was eliminated from the training set during the second classification, etc. The training and test sets are shown in Table 11, and the results of all 13 classifications are presented in Table 12 and Figure 11.

As expected, the greatest deviation was in the first exclusion of the image series. What are the possible causes? The first cut was not uniform. The workpiece does not have an even diameter, therefore conditions were varying along with the cut-the cut depth was not constant. The second, very important aspect is temperature. The workpiece was initially at room temperature. At the first cut, the workpiece was cold, so we infer that it was cooling the insert. Training of the model was executed exclusively on later cuts when both insert and workpiece were already heated, but the first cut was in the test dataset. CNN did not know about any of the images with a cold insert and a workpiece. Besides everything written here, the classification accuracy was 93%, which was well above our expectations.

In later cuts the tool and the workpiece were at least partially heated. When we exclude any other series that followed, the classifications become comparable and are all above 99.6%.

The average classification accuracy for all 13 classifications was 99.39%, although if only series from 2 to 13 are taken into account, it was even higher-at 99.87%. For series from 2 to 13, there were four out of eight incorrect classifications, where an image was one of the first three in the series. It is important that the error is made in random images, and never in two subsequent images. We propose that future system upgrades should include analysis of three subsequent images in the classification and determine wear level only if all three predictions are of the same class. The proposed method would enable the classification accuracy to be 100%.

## 4. Conclusions

Monitoring of the cutting tool wear during the machining process is crucial for final product quality, as well as manufacturing costs’ optimisation. Due to the extreme conditions in the proximity of the cutting (hot chips), in-process tool monitoring becomes difficult. In the scope of the research, we found a solution for equipment protection and developed a method for real-time process monitoring in the immediate proximity successfully.

An IR camera was used, which captures the following process attributes: Visual inspection of the surroundings, workpiece, and chips; acquisition of the temperature conditions and the chip shape. The absolute temperature value was not measured, and the IR camera automatically adjusts the temperature scale for each image. Image recognition is based on the temperature gradients, not on the absolute temperatures.

The research objective was to develop a classification model that would discern the wear level of the cutting insert autonomously, based on deep learning and the convolutional neural network.

First, we categorised the tool condition into four classes (no wear, low wear, medium wear, and high wear). The model was trained using over 8000 images. The test set contained 880 images, out of which 836 were classified correctly. With that, the achieved classification accuracy was 95.00%. Most of the incorrect classifications happened in the classes “medium wear” and “high wear”. For images in series, which were captured from the start of turning (cold tool), the CNN categorised them in the better category rather than the actual. While, after turning for some time (tool and workpiece were heated), some images were classified as a higher wear level than they should be.

It is unusual for industrial machining to distinguish between medium wear level and high wear level. The medium wear level of the insert produces low quality surfaces, also such an insert is overheating and breaks after a short usage time. This model also distinguished between these two categories badly. Based on the classification results and the usefulness for industry we decided that a more sensible categorisation should be into just three classes (no wear, low wear, and high wear). We repeated the classification. Results for the three classes were astounding, because the reached accuracy was 99.55% for a random test set. The average classification accuracy was 99.39% for 13 classifications, where we excluded the image series for the individual test set.

Results were compared with the research which was done by Wu et. al. [27]. Their goal was automatic cutting tool wear type determination (adhesive wear, tool breakage, rake face wear, and flank wear), but they did not determine the adequacy of the worn tool for the machining. They used a classical camera and the CNN for image analysis. The achieved accuracy was 96.20%. The tool classification according to it’s suitability for further machining has already been done by Garcia-Ordas et.al. [28] and achieved accuracy of 90.26% Our system with an IR camera, which was proposed in this article, turned out to be more effective (better accuracy). Its additional benefit is the monitoring of a tool during machining.

The research objective was to develop a model, that makes decisions in place of the human. We were successful in that. With more than 99% model accuracy we affirmed the capability of decision-making about the cutting tool condition, using the IR camera and Artificial Intelligence.

Other smart systems often theoretically determine the type and the size of a tool wear. The presented model makes decisions from a practical point of view-is this tool still suitable for further machining? Practical significance of the research is reliable and fast detection of the tool wear, which ensures savings and an increase of machined surface quality in the industry. The advantage is also a relatively low investment cost, which is estimated at 2500 EUR for all needed equipment.

It was confirmed that the method is suitable for determining the cutting tool condition in a real industrial environment, and that it enables the determination of the tool condition at the first cut.

## 5. Future Work

For industrial use, we propose an additional algorithm, which analyses three subsequent image classifications and determines wear only if all three images belong to the same class. This mitigates the error of random incorrect classifications.

Future work will include an efficiency analysis of the proposed system after the cut (similar to Wu et. al. [27]) and finding the optimal tool condition check time (right after cutting, a few seconds after cutting, etc.).

The experiment was performed under constant cutting conditions. Future work anticipates changing parameters (one by one) and observing which cutting conditions can be changed, no to affect the accuracy of the proposed model. For major changes in cutting conditions (cutting tool, material, etc.) a new learning base should probably be done.

## Figures and Tables

**Table 1:** Methods for determining the level of a cutting tool’s wear.

Direct	Indirect
Visual inspection	Cutting forces
	Chip colour and shape Roughness of a machined surface Vibrations, acoustic emissions Temperature

**Table 2:** Limit values of the wear level.

Wear Level	Dimensional Deviations of the Workpiece
No wear	ΔD < 0.02 mm
Low wear	0.02 mm ≤ ΔD < 0.04 mm
Medium wear	0.04 mm ≤ ΔD < 0.07 mm
High wear	0.08 mm ≤ ΔD

**Table 3:** Machining parameters.

Machining Parameter	Value
Cooling	without
Cutting speed	100 m/min
Feed	0.2 m/min
Cutting depth	0.25 mm (0.5 mm on diameter)

**Table 4:** Chemical compositions (in weight %) of material of the machined workpiece (according to the manufacturer’s specifications; SIJ Metal Ravne, Slovenia).

Mat. No.	EN	DIN	Yield Strength	Chemical Composition %							
				C	Si	Mn	Cr	Mo	Ni	V	W
1.7225	42CrMo4	-	≥900 N/mm^2^	0.41	0.20	0.75	1.05	0.23	-	-	-

**Table 5:** Cutting tool geometric data.

Cutting Edge Length	Relief Angle	Insert Included Angle	Rake	Corner Radius
16 mm	0°	55°	negative	1.0 mm

**Table 6:** The number of images in the training and test sets categorised by the wear class.

	**No Wear**	**Low Wear**	**Medium Wear**	**High Wear**
Training set	2270	1915	1993	2275
Test set	220	220	220	220
TOTAL	2490	2135	2213	2495

**Table 7:** Confusion matrix for a *j*-class classification problem.

		ACTUAL			
		Class 1	Class 2	…	Class *j*
**PREDICTED**	Class 1	TP	FP	FP	FP
	Class 2	FN	TN	TN	TN
	…	FN	TN	TN	TN
	Class *j*	FN	TN	TN	TN

**Table 8:** Confusion matrix for the tool wear classification.

		ACTUAL				Sum	Precision
		No Wear	Low Wear	Medium Wear	High Wear		
**PREDICTED**	No wear	216	1	7	0	224	96.4%
	Low wear	1	219	0	0	220	99.5%
	Medium wear	3	0	194	13	210	92.4%
	High wear	0	0	19	207	226	91.6%
	Sum	220	220	220	220		
	Recall	98.2%	99.5%	88.2%	94.1%		

**Table 9:** Adequacy of the tool according to the machining type.

	Insert Suitable for	
	Rough Turning	Fine Turning
**No wear**	YES	YES
**Low wear**	YES	NO
**High wear**	NO	NO

**Table 10:** Confusion matrix for the classification of the tool wear for three wear categories.

		ACTUAL			Sum	Precision
		No Wear	Low Wear	High Wear		
**PREDICTED**	No wear	218	1	0	219	99.5%
	Low wear	2	219	0	221	99.1%
	High wear	0	0	220	220	100.0%
	Sum	220	220	220		
	Recall	99.1%	99.5%	100.0%		

**Table 11:** Learning and test sets for 13 classifications ^1^.

**Series number**	1	2	3	4	5	6	7	8	9	10	11	12	13
**No. of images in a series**	424	497	498	502	508	505	499	503	487	510	520	486	489
**Classification 1**	T	L	L	L	L	L	L	L	L	L	L	L	L
**Classification 2**	L	T	L	L	L	L	L	L	L	L	L	L	L
**Classification 3**	L	L	T	L	L	L	L	L	L	L	L	L	L
**Classification 4**	L	L	L	T	L	L	L	L	L	L	L	L	L
**Classification 5**	L	L	L	L	T	L	L	L	L	L	L	L	L
**Classification 6**	L	L	L	L	L	T	L	L	L	L	L	L	L
**Classification 7**	L	L	L	L	L	L	T	L	L	L	L	L	L
**Classification 8**	L	L	L	L	L	L	L	T	L	L	L	L	L
**Classification 9**	L	L	L	L	L	L	L	L	T	L	L	L	L
**Classification 10**	L	L	L	L	L	L	L	L	L	T	L	L	L
**Classification 11**	L	L	L	L	L	L	L	L	L	L	T	L	L
**Classification 12**	L	L	L	L	L	L	L	L	L	L	L	T	L
**Classification 13**	L	L	L	L	L	L	L	L	L	L	L	L	T

^1^ L = Learning dataset, T = Test dataset.

**Table 12:** The classification accuracy for each test set.

Tested Image Set	Accuracy	No. of Incorrect Predictions
Series 1	93.63%	27
Series 2	100.00%	0
Series 3	99.80%	1
Series 4	100.00%	0
Series 5	100.00%	0
Series 6	99.80%	1
Series 7	99.80%	1
Series 8	100.00%	0
Series 9	100.00%	0
Series 10	99.61%	2
Series 11	99.62%	2
Series 12	99.79%	1
Series 13	100.00%	0

## Abbreviations

The following physical quantities and abbreviations are used in this manuscript:

ANN	Artificial Neural Networks
BPNN	Backpropagation Neural Network
BUE	Built-Up Edge
CNC	Computer Numerical Control
CNN	Convolutional Neural Network
FN	False Negative
FP	False Positive
GRNN	General Regression Neural Network
IR	Infrared
P	Precision
PMMA	Polymethyl methacrylate
R	Recall
*r*_1_	Actual workpiece radius
*r*_2_	Expected workpiece radius
TN	True negative
TP	True positive
*VB*	Flank wear
Δ*D*	Diameter deviation
